# Deep learning-based image segmentation model using an MRI-based convolutional neural network for physiological evaluation of the heart

**DOI:** 10.3389/fphys.2023.1148717

**Published:** 2023-03-21

**Authors:** Wanni Xu, Jianshe Shi, Yunling Lin, Chao Liu, Weifang Xie, Huifang Liu, Siyu Huang, Daxin Zhu, Lianta Su, Yifeng Huang, Yuguang Ye, Jianlong Huang

**Affiliations:** ^1^ Department of Mathematics and Computer Science, Quanzhou Normal University, Quanzhou, China; ^2^ Fujian Provincial Key Laboratory of Data Intensive Computing, Quanzhou, China; ^3^ Key Laboratory of Intelligent Computing and Information Processing, Fujian Province University, Quanzhou, China; ^4^ Department of General Surgery, Huaqiao University Affiliated Strait Hospital, Quanzhou, China; ^5^ Department of Diagnostic Radiology, Huaqiao University Affiliated Strait Hospital, Quanzhou, China

**Keywords:** cardiac MRI, image segmentation, U-Net, batch normalization layer, physiological analysis

## Abstract

**Background and Objective:** Cardiovascular disease is a high-fatality health issue. Accurate measurement of cardiovascular function depends on precise segmentation of physiological structure and accurate evaluation of functional parameters. Structural segmentation of heart images and calculation of the volume of different ventricular activity cycles form the basis for quantitative analysis of physiological function and can provide the necessary support for clinical physiological diagnosis, as well as the analysis of various cardiac diseases. Therefore, it is important to develop an efficient heart segmentation algorithm.

**Methods:** A total of 275 nuclear magnetic resonance imaging (MRI) heart scans were collected, analyzed, and preprocessed from Huaqiao University Affiliated Strait Hospital, and the data were used in our improved deep learning model, which was designed based on the U-net network. The training set included 80% of the images, and the remaining 20% was the test set. Based on five time phases from end-diastole (ED) to end-systole (ES), the segmentation findings showed that it is possible to achieve improved segmentation accuracy and computational complexity by segmenting the left ventricle (LV), right ventricle (RV), and myocardium (myo).

**Results:** We improved the Dice index of the LV to 0.965 and 0.921, and the Hausdorff index decreased to 5.4 and 6.9 in the ED and ES phases, respectively; RV Dice increased to 0.938 and 0.860, and the Hausdorff index decreased to 11.7 and 12.6 in the ED and ES, respectively; myo Dice increased to 0.889 and 0.901, and the Hausdorff index decreased to 8.3 and 9.2 in the ED and ES, respectively.

**Conclusion:** The model obtained in the final experiment provided more accurate segmentation of the left and right ventricles, as well as the myocardium, from cardiac MRI. The data from this model facilitate the prediction of cardiovascular disease in real-time, thereby providing potential clinical utility.

## 1 Introduction

Currently, in the medical domain, various medical imaging techniques and advanced equipment are utilized (Hu et al., 2019), including computed tomography, X-ray, and magnetic resonance imaging. As a new medical imaging diagnostic technology, MRI has developed very rapidly in recent years. MRI not only provides more information compared to many other imaging techniques but also has significant potential advantages in disease diagnosis. Improvement in medical imaging technology is closely related to advancements in computer technology (Lou et al., 2020). The use of computer technology in the domain of medical imaging enables doctors to see pathological tissue structures more easily, improves the efficiency of disease analysis, and provides more accurate medical reports, thereby greatly reducing the rate of misdiagnosis (Olaf et al., 2015).

We have proposed a series of methods for cardiac image segmentation based on our deep-learning algorithm. Avendi et al. (2016) proposed a method to combine an algorithm and a learning deformation model for the segmentation of the left ventricle. Because the left ventricle tends to contract easily and is sensitive to initialization, this method automatically uses the convolutional neural network to detect the left ventricular chamber. The accuracy of the segmentation is increased by using the stacked automatic encoder to determine the left ventricular chamber based on the data model and the combination of the anticipated shape to develop the formable model. van der Geest and Reiber (1999) provided a method for segmenting the left ventricle using a deep learning model along with several level sets. Based on the shape and appearance of the training set, it required less training data, but it had limitations when the region of interest in the training set was modeled differently. The authors combined the benefits of both methods to achieve left ventricular segmentation using deep learning, thereby simulating this shift with less annotated training sets, yet usually involving regularization to enhance the generalization ability (Sulaiman et al., 2018).

Segmentation accuracy has improved through the use of deep learning-based models compared with traditional methods for dividing heart images (Geert et al., 2017). However, the models still lack the capability to completely differentiate the left and right ventricles from the myocardium, and the training complexity is high. To enhance the performance of the neural network, this study adopted the improved U-Net network in a fully convolutional neural network to conduct cardiac segmentation. Our study also demonstrates the addition of batch normalization (BN) and the adoption of different loss functions. Finally, we conducted an experimental evaluation of the data set and achieved superior segmentation results compared to previous work by applying the batch normalization layer and the combination weighted loss function.

## 2 Materials and methods

### 2.1 Network topology

**FIGURE 1:**
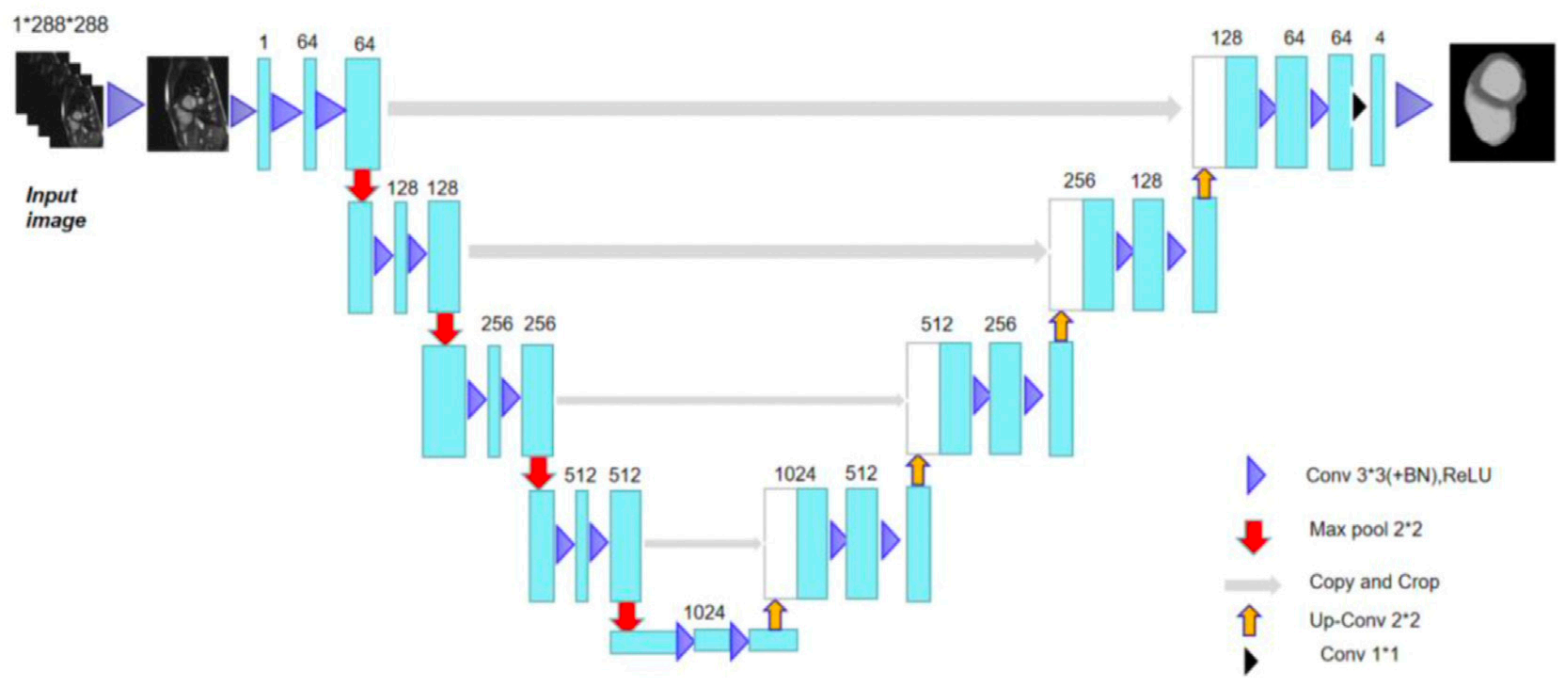
Classic U-network structure for the purpose of cardiac MRI image segmentation.

In this study, the network adhered to the traditional U-Net network (Sulaiman et al., 2018) encoding and decoding structure (Olaf et al., 2015), and Figure 1 depicts the network model structure (Wong et al., 2020). The network learned how to encode the image characteristics in the training set along the encoding route.

The network obtained image characteristics from the acquired encoding characteristics in the decoding route (Shi et al., 2021) and reconstructed the images. The cascade between the outputs of each stage and the inputs of the decoding path defined the U-Net network topology. The convolution network of the decoding path was where these cascade operations identified the high spatial resolution information on the image. Therefore, using these data as a foundation, a more accurate result was produced. The encoding route had two identical sets of 33 convolutions at each stage. Improvement of the U-Net (Zhang et al., 2018) could be achieved by adding every 33 convolutions and a rectified linear unit (ReLU) (Li et al., 2020) of the network. Each layer was used for subsampling to a maximum pooling layer with 22 convolution steps of 2. The network predetermined 64 feature channels to be the starting number. The image size was reduced by half and the number of feature channels was doubled following the subsampling of each layer (Wong et al., 2017).

There were two identical 33 convolutions included in each step of the upsampled decoding method. Similar to this, a batch normalizing layer and a ReLU were applied after each convolution. With 22 convolution upsampling, the number of feature channels was halved and the upsampled feature images cascaded with the feature images from the encoded path. A total of 64 feature maps could be mapped to the four classes of heart segmentation by adding an additional 11 convolution layers to the final layer (the left ventricle, myocardium, right ventricle, and background). In other words, the output of the last layer suggested that one of the four classes following the softmax classifier was where the pixels belonged.

### 2.2 Function of batch normalization

To create a Gaussian distribution with a mean of 0 and a variance of 1, normalization (Anwar et al., 2018) was used to change the input data. The normalized layer has the capacity to return the output data distribution of the preceding layer to its initial state throughout the training cycle. To avoid forcing the network to adapt to a new data distribution, the processed data were fed into the network’s subsequent layer, which took the initial state’s data distribution as input (Su et al., 2019). High learning rates can be used during network training to hasten network convergence and reduce network overfitting. The batch normalization layer keeps each layer’s size constant and in line with the dimension. A batch of data is supplied into the training phase once the linear activation unit has been standardized, where *m* is the amount of data.

As the first step, the data means for this batch were calculated as follows:

$$\mu_{B}=\frac{1}{m}\overset{m}{\underset{i=1}{\sum }}x_{i}.$$ (1)

Then, the variance was given by

$$\sigma_{B}^{2}=\frac{1}{m}\overset{m}{\underset{i=1}{\sum }}x_{i}{\left(x_{i}-\mu_{B}\right)}^{2}.$$ (2)

After standard processing,

$${\hat{x}}_{i}=\frac{x_{i}-\mu_{B}}{\sqrt{\sigma_{B}^{2}-\in }}.$$ (3)

Here, for an integer with very small values, we needed to avoid the value of $\sigma_{B}^{2}$ being 0.

The data distribution was only allowed to follow a Gaussian distribution after standardization processing, while the data distribution for some layers was not. The capacity of the network for learning will be impacted by such standardization operations. With the addition of new scaling parameters *γ* and translation parameters *β*,

$$y_{i}=\gamma {\hat{x}}_{i}+\beta =BN_{\gamma ,\beta }\left(x_{i}\right).$$ (4)

The newly added parameters, *γ* and *β*, are a pair of learnable parameters that take part in network training and can restore the distribution of features that the original network needs to learn.

### 2.3 Training the loss function with the network model hyperparameters

In this study, three loss functions to train improved U-Net networks were used. To compute the difference between background and foreground class, each pixel was individually inspected for class prediction compared to the target vector encoded by one-hot using the pixel-wise cross-entropy loss function (Diederik and Jimmy, 2014).

In the experiment, the network training incorporated the Dice coefficient with the aim of obtaining a relatively stable gradient. When calculating the Dice loss in the training network softmax output layer, the Dice was reduced by 1, and the coefficient obtained by the Dice loss function was

$$L_{diceloss}=1-2\frac{A\cap B}{\left|A\right|+\left|B\right|+ò},$$ (5)

where *B* represents the outcome after output through the softmax layer, which is a constant set close to 0, i.e., $e^{-10}$ , and *A* represents the real value (ground truth). The loss trends toward zero, and the loss function converges as the Dice coefficient gets closer to 1.

When training multi-class targets, the following pixel-level loss function L is often introduced:

$$L=-\overset{M}{\underset{j=1}{\sum }}y_{j}\log P_{j},$$ (6)

where *M* is the number of categories, *P*_*j*_ is the *j*th value of the output *P* of softmax (*j* = 1, …, *M*) that indicates the probability that the sample falls into class *j*, and *y*_*j*_ is the correct label (using one-hot, when *j* is a certain class, the index value of the correct label in *y*_*j*_ is 1, and the others are 0).

This study combines pixel-level loss function and Dice loss function as follows:

$$L_{crossentropydice}=\alpha L+\beta L_{diceloss},$$ (7)

where *L* is the pixel-level loss function, *L*_*diceloss*_ is the Dice loss function, and *α* and *β* are the respective weights, *α* = 1 and *β* = 0.2.

We utilized the Adaptive Moment Estimation (ADAM) optimizer and implemented a batch size (one set of training data) of 4, 18 epochs (training rounds), and a learning rate of 0.001 to decrease the loss function.

### 2.4 Experimental data acquisition

The short-axis cardiac MRI experimental data were obtained from the Huaqiao University Affiliated Strait Hospital. All patients in this study had given written consent for participation in the study. Ethical approval was granted by the review committee of our institutional board. The medical image dataset had a total of 275 MRI scans of hearts that consisted of two stages, which pertain to the end-diastole and end-systole. The spatial resolution range of this dataset was 0.70 mm × 0.70 mm–1.92 mm × 1.92 mm. We divided the data into training and test sets using an 8:2 ratio.

**FIGURE 2:**
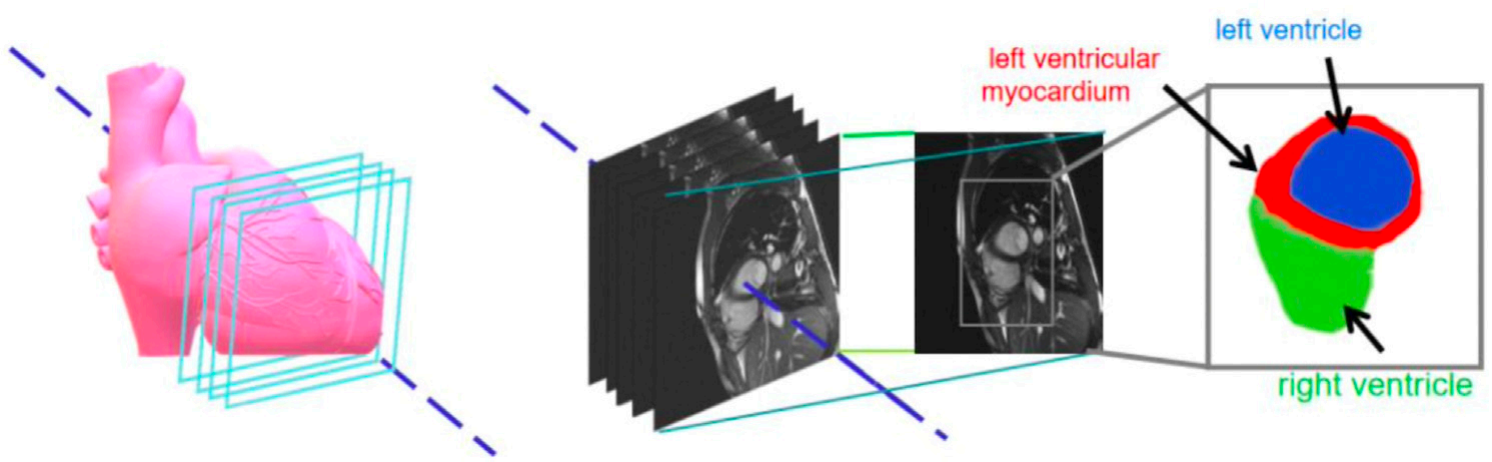
Sample datasets pertaining to cardiac images and magnetic resonance images with respective enlargements.

The gold standard image contained three-pixel regions: green representing the left ventricle; red, the left ventricular myocardium; and blue, the right ventricle. Figure 2 shows the original image of a patient’s heart section and the corresponding gold standard. The number of images in the data set needed to be increased. The data enhancement method was adopted to expand the number of experimental images by random cropping, rotation, and flipping operations. The data enhancement operation was carried out in the network training to reduce data storage (Olaf et al., 2015; Hao et al., 2017; Ye et al., 2021).

### 2.5 Evaluation indicators

Three assessment coefficients [Dice coefficient, Hausdorff distance (HD) (Khened et al., 2019), and average symmetric surface distance (ASSD)], which are frequently employed to gauge the effectiveness of segmentation, were utilized to assess the similarity of segments and label the images.

The degree of similarity between the two segmentation results and the reference image was gauged by the Dice coefficient, which is given by

$$Dice\left(A,B\right)=2\frac{\left|A\cap B\right|}{\left|A\right|+\left|B\right|},$$ (8)

where *A* represents the true value and *B* represents the following range pertaining to the softmax structure’s output, which is based on the heart image segmented by the network structure and measures the similarity between the two segmentation outputs and the reference image. The Dice coefficient is a number between 0 and 1. When the Dice value is close to 1, it indicates that the heart image has been segmented well; when it is close to 0, it indicates that the heart image has been poorly segmented and there is little overlap with the gold standard image.

The Hausdorff distance is the greatest distance between two points in another set and is calculated as follows:

$$H\left(A,B\right)=\left(\max_{a\in A}\left(\min_{b\in B}\left(d\left(a,b\right)\right)\right),\max_{b\in B}\left(\min_{a\in A}\left(d\left(a,b\right)\right)\right)\right),$$ (9)

where *A* stands for the idealized image, *B* for the segmented image, and *d* (*a*, *b*) for the Euclidean distance.

The average symmetric surface distance is a metric based on the surface distance, referring to the distance between the surface pixel *S* (*A*) of the gold standard *A* and the surface pixel *S* (*B*) of the segmentation result *B,* and calculated as follows:

$$ASD\left(A,B\right)=\frac{1}{S\left(A\right)+S\left(B\right)}\left(\underset{S_{A}\in S\left(A\right)}{\sum }d\left(S_{A},S\left(B\right)\right)+\right.\left.\underset{S_{B}\in S\left(B\right)}{\sum }d\left(S_{B},S\left(A\right)\right)\right),$$ (10)

where *A* represents the standard image, *B* represents the model results, and *S* (*A*) and *S* (*B*) represent the set of surface pixels of *A* and *B*, respectively. *d* (*SA, S* (*B*)) is a representation of the shortest Euclidean distance between each point on *S* (*A*) and all other points on *S*.

## 3 Results

### 3.1 Comparison of test results for networks trained using different loss functions

The network was trained with different kinds of loss functions, and left ventricle, right ventricle, and myocardium segmentation results were evaluated with the Dice coefficient and average symmetric surface distance. After averaging the two phases, we reached the final evaluation shown in Table 1. The least accurate experimental results were obtained using a Dice loss function, and the best were obtained using a weighted combined loss function combining Dice loss with a pixel-level loss function. Usually, the Dice loss function will have a negative effect on backpropagation, which renders the training unstable but allows learning from the smaller classes in the image; the pixel-level cross entropy function is used for multiple classification tasks, but it is susceptible to the categories with more pixels, thereby making it difficult to learn the characteristics of categories with fewer pixels. Thus, merging both advantages, this combined weighted loss function has been considered as the best performing loss function for experimental network training. The test results from networks trained using a combined weighted loss function network are presented in Table 1.

**TABLE 1:** Comparison of mean segmentation accuracy of three loss functions based on improved U-Net network.

	Left ventricle		Right ventricle		Myocardium	
	Dice	ASSD	Dice	ASSD	Dice	ASSD
Dice loss	0.93	0.61	0.82	2.40	0.88	0.56
Cross entropy	0.93	0.69	0.86	1.60	0.88	0.64
L_Crossentropy_	0.94	0.53	0.89	1.03	0.89	0.51

### 3.2 Test results from networks trained using a combined weighted loss function network

The improved U-Net network (Zhao et al., 2020) used a new combined weighted loss function, and the resulting network model was used to distinguish the left ventricle and right ventricle from the ED stage to the ES stage. The statistical segmentation accuracy values and statistical results are presented in Table 2. The data shows that the highest value for the left ventricle occurred in the ES stage and the lowest in the ES stage.

**TABLE 2:** Comparison of segmentation accuracy values based on improved U-Net network.

	Left ventricle			Right ventricle			Myocardium		
	Dice	HD	ASSD	Dice	HD	ASSD	Dice	HD	ASSD
ED	0.965	5.40	0.37	0.938	11.75	0.58	0.889	8.34	0.48
ES	0.926	6.92	0.70	0.860	12.66	1.49	0.901	9.29	0.54
Mean	0.942	6.16	0.53	0.899	12.20	1.03	0.895	8.82	0.51

**FIGURE 3:**
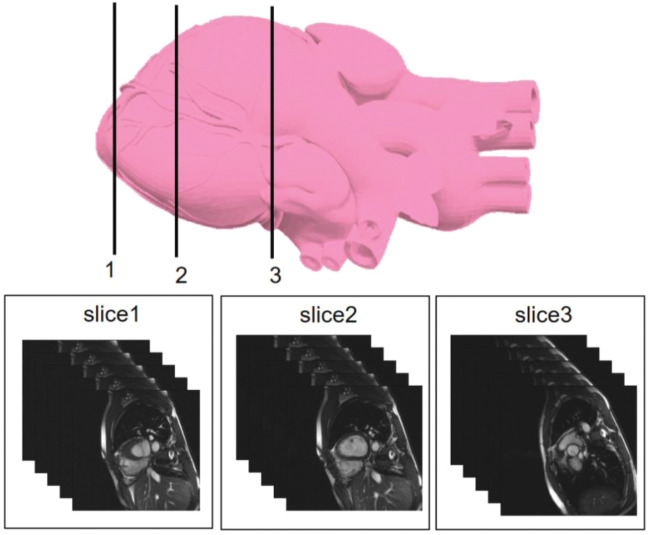
Cardiac MRI sampling locations included in this study.

**FIGURE 4:**
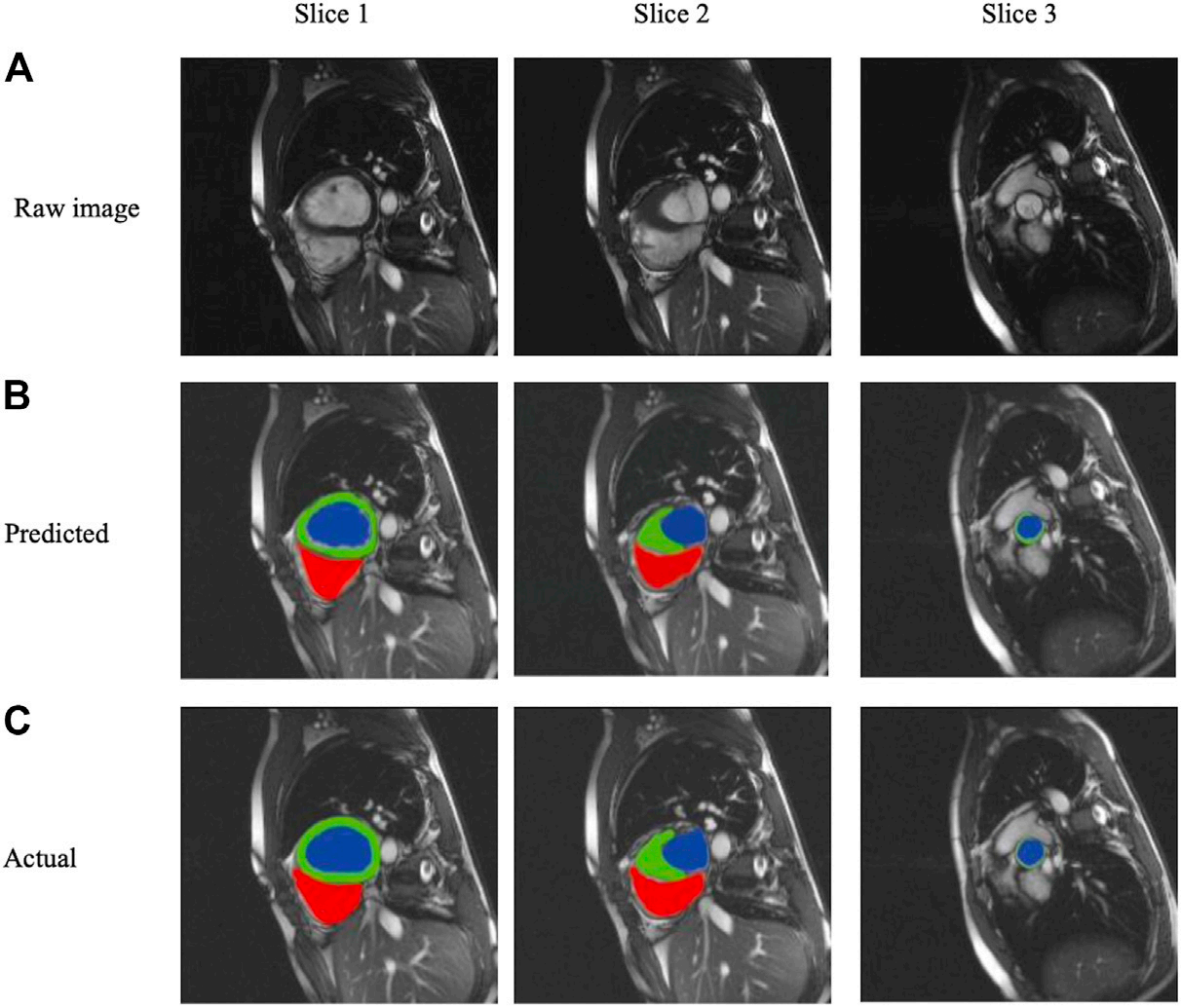
Segmentation results from analysis of the three slice heart samples based on **(A)** raw images, **(B)** Predicted segmentation images, and **(C)** Actual gold standard segmentation images; red areas represent the right ventricle, green areas represent the myocardium, and blue areas represent the left ventricle.

**FIGURE 5:**
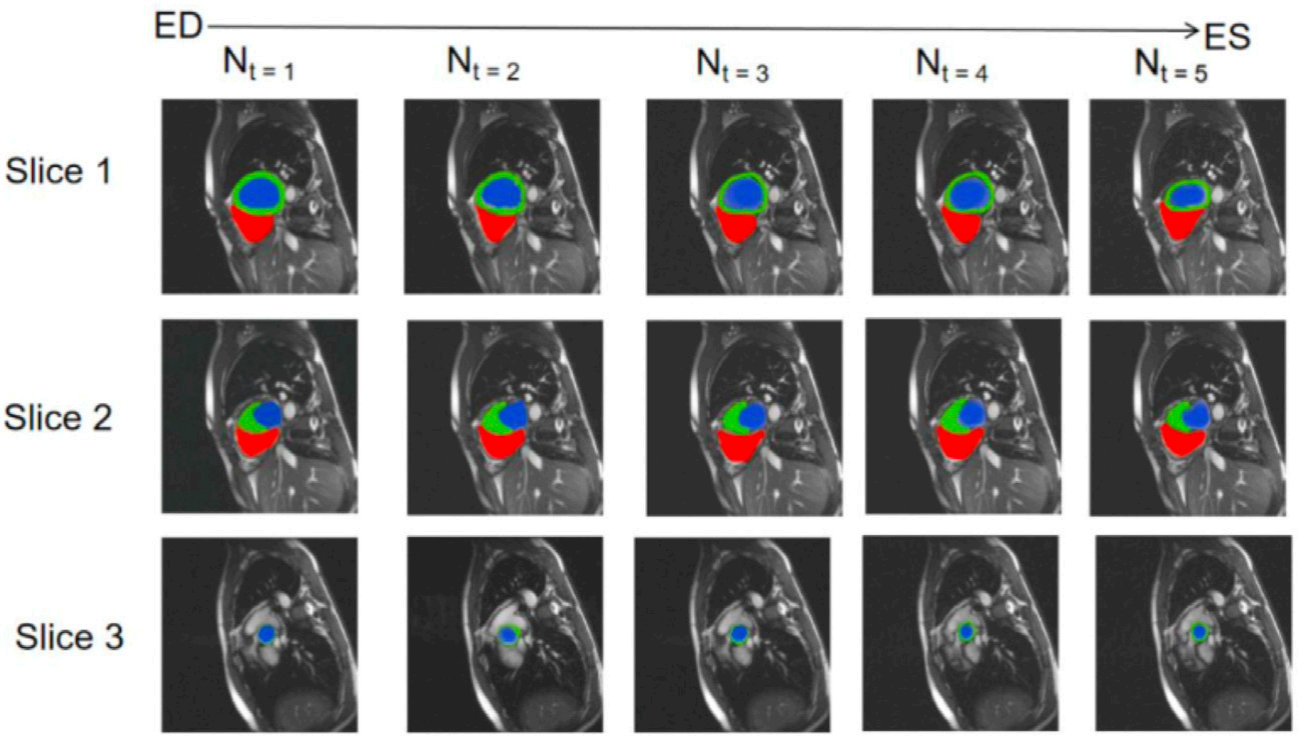
Predicted segmentation from stage ED to stage ES; red areas represent the right ventricle, green areas represent the myocardium, and blue areas represent the left ventricle.

We selected three representative sections from the data for demonstration, as shown in Figure 3. From left to right, we obtained sections of the lower heart, middle heart, and upper heart, which are depicted as Slice 1, Slice 2, and Slice 3, respectively. Figure 4 shows the segmentation results of our modified U-Net network in the test set. From upper to lower in position, the rows represent the original image, segmentation result, and corresponding true value. Figure 5 shows the segmentation predicted from stage ED to stage ES. The red area represents the right ventricle, the green area represents the myocardium, and the blue area represents the left ventricle. The second row reveals that the method showed more accurate segmentation results in the analysis of the data set representing the middle of the slice, but the segmentation of the base and the top of the slice was poor due to the top of the heart slice containing very few ventricular pixels, resulting in the ventricular boundary being too difficult to confirm as ventricular density was close to tissue interference, etc.

### 3.3 Comparison of results by other methods

The experimental method was compared with different segmentation methods (Simonyan and Zisserman, 2014), whereby each method of heart data set segmentation was evaluated using the Dice coefficient and Hausdorff distance. Segmentation results of the left and right ventricular and myocardial accuracy values based on the two evaluation indices are presented in Table 3. From the experimental segmentation results compared with other methods, the Hausdorff distance value was the lowest, and the best performance occurred in the segmentation of the myocardium in the ED stage, demonstrating that the Hausdorff distance value was the lowest. These data show that the experimental training network improved the network segmentation, which indicates that this experimental method has certain advantages and results in much-improved segmentation of the heart.

**TABLE 3:** Segmentation accuracy statistics of different segmentation methods.

	Methods	Literature	Dice		Hausdorff	
			ED	ES	ED	ES
LV	U-Net	Isensee (Isensee et al., 2017) and Jaeger (Isensee et al., 2017)	0.968	0.931	7.4	6.9
	Modified U-Net + loss function	Zotti (Zotti et al., 2018) and Luo (Zotti et al., 2018)	0.963	0.912	6.2	8.4
	2D U-Net and 3D U-Net	Baumgartner (Baumgartner et al., 2017)	0.963	0.911	6.5	9.2
	2D FCN	Jang (Jang et al., 2017), Hong (Jang et al., 2017), and Ha (Jang et al., 2017)	0.959	0.921	7.7	7.1
	FCN + U-Net + BN	Current study	0.957	0.911	5.7	6.7
RV	U-Net	Isensee (Isensee et al., 2017) and Jaeger (Isensee et al., 2017)	0.946	0.899	10.1	12.2
	Modified U-Net + loss function	Zotti (Zotti et al., 2018) and Luo (Zotti et al., 2018)	0.934	0.885	11.1	12.7
	2D U-Net and 3D U-Net	Baumgartner (Baumgartner et al., 2017)	0.932	0.883	12.7	14.7
	2D FCN	Jang (Jang et al., 2017), Hong (Jang et al., 2017), and Ha (Jang et al., 2017)	0.929	0.885	12.9	11.8
	FCN + U-Net + BN	Current study	0.932	0.862	11.5	12.7
Myo	U-Net	Isensee (Isensee et al., 2017) and Jaeger (Isensee et al., 2017)	0.902	0.919	8.7	8.7
	Modified U-Net + loss function	Zotti (Zotti et al., 2018) and Luo (Zotti et al., 2018)	0.886	0.902	9.6	9.3
	2D U-Net and 3D U-Net	Baumgartner (Baumgartner et al., 2017)	0.892	0.901	8.7	10.6
	2D FCN	Jang (Jang et al., 2017), Hong (Jang et al., 2017), and Ha (Jang et al., 2017)	0.882	0.897	9.8	11.3
	FCN + U-Net + BN	Current study	0.887	0.923	8.5	9.5

## 4 Discussion

We trained a new model for cardiac segmentation from MRI data learning and effectively improved experimental results by adding batch normalization layers to the neural network and using different loss functions. Compared with the existing deep learning segmentation method (Olaf et al., 2015; Avendi et al., 2016), we used the improved U-Net network in the FCN Network for cardiac MRI segmentation, added BN, and selected different loss functions to improve the neural network performance. Good segmentation results were obtained by combining the batch normalization layer and the integrated weighted loss function. At the same time, due to the tendency of the heart to contract easily, the method is sensitive to initialization and other problems. Research using myocardial cell models or cardiac-related cells of the ventricular structures has combined analytics to measure intimate cellular interactions (Wong, 2017), such as those at the apical segment, which have been found to have a strong influence on dynamical changes or even thickening of the ventricular wall; these data may provide new insights into cardiovascular structure dynamics. We referred to the convolutional neural network detailed in existing research (Su et al., 2019) to automatically detect the left ventricle in the dataset and, at the same time, the stacked autoencoder was used to judge the left ventricular chamber. The method of merging these aspects into a formable model solved a series of problems, including detail extraction. However, we have not yet been able to achieve complete segmentation of the heart ventricle and the atrium (Chen et al., 2020; Zhu et al., 2021; Wong et al., 2022), or that of the aorta (Wong et al., 2006; Chen et al., 2022), which will be the focus of our upcoming research directions toward future implementation.

## 5 Conclusion

Through our experiments, we trained a deep learning model with the capacity to achieve heart segmentation based on MRI data by adding a batch-normalized layer to the neural network and using different loss functions and numbers, which improved the experimental results. The model described in this study has been improved in the algorithmic sense. In subsequent studies, other network structures may be added to the U-Net network’s downsampling process to obtain a better network downsampling structure. Additionally, the loss function selected by the network greatly influenced the experimental results; therefore, the design of a new loss function may further improve experimental accuracy.

## Data Availability

The original contributions presented in the study are included in the article/Supplementary Material, further inquiries can be directed to the corresponding authors.
